# Troublesome toxins: time to re-think plant-herbivore interactions in vertebrate ecology

**DOI:** 10.1186/1472-6785-9-5

**Published:** 2009-02-24

**Authors:** Robert K Swihart, Donald L DeAngelis, Zhilan Feng, John P Bryant

**Affiliations:** 1 Department of Forestry and Natural Resources, Purdue University, West Lafayette, IN 47907, USA; 2 US Geological Survey and Department of Biology, University of Miami, Coral Gables, Florida 33124, USA; 3 Department of Mathematics, Purdue University, West Lafayette, IN 47907, USA; 4 Box 306, Cora, Wyoming 82825, USA

## Abstract

Earlier models of plant-herbivore interactions relied on forms of functional response that related rates of ingestion by herbivores to mechanical or physical attributes such as bite size and rate. These models fail to predict a growing number of findings that implicate chemical toxins as important determinants of plant-herbivore dynamics. Specifically, considerable evidence suggests that toxins set upper limits on food intake for many species of herbivorous vertebrates. Herbivores feeding on toxin-containing plants must avoid saturating their detoxification systems, which often occurs before ingestion rates are limited by mechanical handling of food items. In light of the importance of plant toxins, a new approach is needed to link herbivores to their food base. We discuss necessary features of such an approach, note recent advances in herbivore functional response models that incorporate effects of plant toxins, and mention predictions that are consistent with observations in natural systems. Future ecological studies will need to address explicitly the importance of plant toxins in shaping plant and herbivore communities.

## The importance of plant-herbivore interactions

By definition herbivores depend on plants to survive. The need to obtain suitable food in sufficient amounts drives innumerable herbivore behaviors; for example, movement decisions often are related to the distribution and abundance of plant resources [1]. By the same token, herbivores can exert strong effects on plant growth, survival, and population size by virtue of their feeding habits. Plant demographic effects are especially severe during cyclical peaks or irruptions in herbivore populations [2,3]. Moreover, the ecological effects of herbivores can extend beyond populations. Differential foraging among species can affect outcomes of competition, facilitate invasion of extant communities, and alter patterns of plant succession, diversity, and dominance [4-6].

## Conventional modeling approaches

When focusing on optimal diet choice by herbivores, ecologists traditionally have relied on linear programming or linear dynamic programming methods [7,8]. Given a choice of two or more non-equivalent food types, these methods solve for optimal diet composition subject to constraints imposed by daily energy requirements, feeding time, digestive capacity, or nutrient requirements. Linear programming appears to provide reasonable predictions of diet composition for many species [9]. However, it does not address population-level dynamics of herbivores and plants.

Consumer-resource interactions at the population level can be modeled using equations that relate the rate of resource intake by a consumer to resource abundance [10]. These so-called "functional-response" models link herbivore behavior and plant characteristics to population- and community-level consequences. In these models, upper limits to rates of consumption by herbivores are determined, either implicitly or analytically, by combining mechanical factors such as bite size and rate with plant quantity [11-13].

## Ignore plant toxins

A problem with conventional plant-herbivore models is their failure to incorporate factors related to plant quality into decelerating functional responses. For many herbivores, plant toxicity plays an important role in diet choice [14,15]. Indeed, plants in both tropical and temperate systems appear to have evolved a variety of chemical defenses, many of which are unique to particular plant species [16]. For instance, many Australian *Eucalyptus *trees produce 1,8-cineole, a monoterpene that serves as a potent deterrent to herbivorous marsupials such as brushtail possum, *Trichosurus vulpecula *[17]. Creosote bush (*Larrea tridentata*) in the western United States produces phenolic resins containing nordihydroguaiaretic acid, which limits intake by desert woodrats, *Neotoma lepida *[18]. Tree birches (*Betula*) in boreal North America produce the triterpene papyriferic acid as a deterrent to feeding by snowshoe hares, *Lepus americanus *[19]. Although most work on chemical defenses against vertebrate herbivores has involved mammals [14], plant toxins also influence herbivorous birds. For instance, aspen (*Populus tremuloides*) produces coniferyl benzoate, a phenylpropanoid ester that inhibits feeding by ruffed grouse, *Bonasa umbellus *[20].

In addition to interspecific differences, production of toxins varies ontogenetically within plants, and among individuals and populations within species. Intraspecific variation in chemical defense often contains strong genetic components [21-23]. When combined with spatial variation in environmental conditions and herbivory, substantial geographic variation in defense can occur within species [24,25]. Ontogenetic variation in defensive responses of many plants is shaped by constraints on resource allocation and sensitivity to fitness consequences of herbivory [26]. For instance, winter browsing of plants by mammals has severe repercussions for fitness during the juvenile stage and was linked to greater defense of juveniles in a review of 37 woody species [27].

## And coping strategies of herbivores

One consequence of feeding on plants containing toxins is that rates of ingestion may be limited by an herbivore's ability to avoid toxins or detoxify food rather than to mechanically process food. Not surprisingly, herbivores have developed a host of physiological and behavioral mechanisms to deal with plant secondary metabolites [28]. Physiologically, vertebrates can regulate absorption of plant toxins by gut cells, respond to chemically mediated taste and trigeminal stimulation, and detoxify lipophilic compounds via enzymatic biotransformation [14]. For instance, marsupial folivores oxidize plant terpenes using P450 enzymes, and species with diets high in monoterpenes exhibit greater capacity for biotransformation of toxins than their generalist counterparts [29]. Behaviorally, vertebrates can select plants or plant parts containing low concentrations of a toxin [30], manage food to leach toxins from plants [31,32], self-medicate to ameliorate effects of toxins [33], and adjust meal duration and intake per meal [34,35]. An ability to regulate intake of plant secondary metabolites has been reported for several species of vertebrate herbivores [17,34,36]. For instance, brushtail possums ate more of the toxin benzoate when the rate at which it could be detoxified by conjugation was increased by adding glycine to the diet [37]. Herbivores also achieve greater intake of nutrients by selecting mixed diets containing foods processed by different detoxification pathways, thereby avoiding saturation of any particular pathway [38,39]. Regardless of the strategies used by herbivores, costs of detoxification often are high. For desert woodrats subsisting on a diet containing a toxin-rich juniper (*Juniperus monosperma*), detoxification costs are comparable to energy needed for reproduction [40]. For ruffed grouse feeding on aspen, 10 percent of metabolizable energy is lost each day in biotransformation conjugates; additional losses of energy in the conjugation process and of nitrogen due to excretion of amino acid conjugates elevate the cost further [36]. In the face of such costs, vertebrate herbivores face life-history tradeoffs associated with allocation of resources to growth and reproduction [41].

## Needed: A toxin-determined functional response

In light of the widespread nature of plant toxins and their influence on herbivores, a new approach to linking herbivores to their food base is needed if we are to understand implications for herbivore populations and plant communities. Traditional functional responses for vertebrate herbivores have not considered the role of plant toxins. At least three analytical modifications should be considered when incorporating the effects of toxins on plant-herbivore dynamics. Toxin-mediated functional responses should (1) explicitly account for the negative effects of plant toxins on herbivore growth; (2) permit herbivores to regulate intake of toxins; and (3) allow for intake of multiple plants that are detoxified with independent pathways. Recently, progress has been made in the first two areas [42,43]. Specifically, a conventional functional response has been modified [43,44] to take the form of *C(N)*:

(1) $$C(N)=f(N)\left(1-\frac{f(N)}{4G}\right),$$

where

$$f(N)=\frac{e\sigma N}{1+he\sigma N}.$$

The term *f(N) *is the traditional Holling Type 2 functional response in which *N *is plant biomass, *e *is resource encounter rate, *h *is handling time for each plant, and *σ *is the fraction of encountered food items that are ingested, thereby allowing herbivores to regulate their intake. The second factor in *C(N) *explicitly accounts for the negative effect of toxins. The parameter *G *stands for the ratio *M/T*, where *M *is the maximum amount of toxicant per unit time that the herbivore can tolerate and *T *is the amount of toxicant per unit of plant biomass. The factor 4 simplifies the peak value of *C(N) *as a function of *N*. In the limit that 1*/G *<< 1, the effect of the toxicant can be viewed as purely a slowdown in feeding rate. For example, in that limit *C*(*N*) above is an approximation of

(2) $$C(N)≅\frac{e\sigma N}{1+he\sigma N+\frac{e\sigma N}{4G}}$$

So in (2), the presence of toxin simply results in an effective increase in the handling time that is proportional to 1/*G*.

Related functional response models have been formulated to examine how plant growth is limited when the presence of one resource interferes with another resource or is toxic [45]. In other models, additive effects of nutrient limitation of plant growth have been incorporated [46]:

(3) $$C=\frac{C_{\max}N_{1}}{K_{1}+N_{1}+K_{2}\frac{N_{1}}{N_{2}}},\text{ where}$$

*N*_1 _and *N*_2 _represent the concentrations of nutrients limiting plant growth, C_max _is the maximum possible rate of nutrient-limited plant growth, and *K*_1 _and *K*_2 _are constants reflecting the stoichiometry of the two nutrients in the plant. Note that in the limiting case, as *N*_1_/*N*_2 _approaches zero, (3) reduces to the traditional Michaelis-Menten equation for *N*_1_, with *K*_1 _as a half-saturation coefficient [47]. More importantly from our perspective, increasing availability of the co-limiting nutrient, *N*_2_, causes growth rate to increase in (3), whereas in (2) an increasing concentration of toxin in food relative to the rate of toxin ingestion the herbivore can tolerate, *1/G*, decreases growth rate. In both equations, this change in *C *is due to a change in size of the third term in the denominator.

The toxin-determined functional response (1) differs from plant-nutrient models (3) because toxins can do more than reduce feeding rate. Specifically, when 1*/G *is large (and hence each gram of plant is quite toxic to herbivores), the functional response can represent a more serious deterioration of the herbivore's ability to feed or survive. Analysis has demonstrated the critical importance of *σ *to herbivore dynamics; in the presence of a toxin, selection should act strongly to regulate intake below the herbivore's detoxification threshold [43].

**Figure 1:**
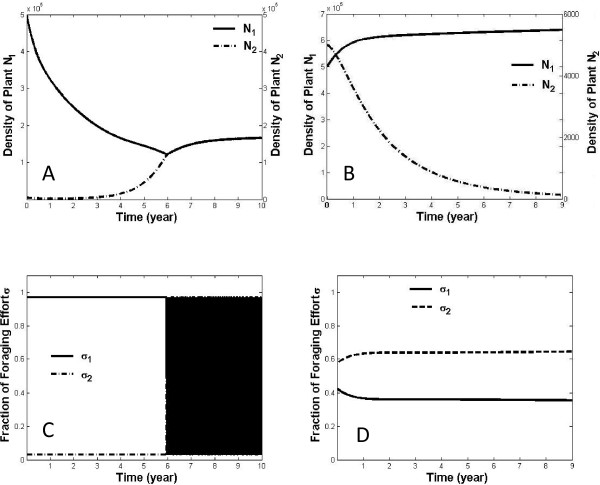
**Dynamics of two-species plant communities subjected to a population of herbivores that feed optimally**. *N*_1 _and *N*_2 _refer to biomasses of edible plants. The functional response used to generate the plots is the one shown in equation (1) of the text, modified to permit two plant species [48]. When toxins are not incorporated into the functional response (panel A), the plant species coexist. Note that herbivores feed exclusively on plant species 1 initially, i.e., *σ*_1 _= 1, *σ*_2 _= 0 (panel C), since the starting density of species 1 is higher. Once the density of species 2 exceeds that of species 1, the consumption constants switch to *σ*_1 _= 0, *σ*_2 _= 1, and the switches continue to occur (the switches occur so rapidly that it appears as a black area in panel C). When toxins are incorporated into the functional response and the resident plant species (species 1 in panel B) is more toxic than a prospective invading species (species 2 in panel B), simulation results demonstrate that the less toxic plant fails to establish. The failure is tied to the adaptive foraging behavior of the herbivore, resulting in a disproportionate fraction of its effort being expended on the less abundant (but less toxic) species 2 (panel D). Parameter values: *c*_12 _= 0.9, *c*_21 _= 0.9, *r*_1 _= *r*_2 _= 0.007, *K*_1 _= *K*_2 _= 7*10^5^, *B*_1 _= *B*_2 _= 3.4*10^-5^, *e*_1 _= *e*_2 _= 0.0007, *h*_1 _= *h*_2 _= 0.008, *m*_p _= 0.0013. For simulations of plants containing toxins (panels B and D): *G*_1 _= 35, *G*_2 _= 60, initial density of species 1 = 5 × 10^5^, initial density of species 2 = 5 × 10^3^.

Because most vertebrate herbivores are generalists, a toxin-mediated functional response should be able to consider multiple plant species. Fortunately, the single-species framework [43] extends directly to multiple species. Recent analysis of a multi-species model with independent pathways for detoxification [48] yielded predictions that matched remarkably well with empirical studies of moose (*Alces alces*) and snowshoe hares feeding on woody plants. Under conditions of the multi-species model, herbivores switch feeding on plant species to avoid saturating detoxification systems. One consequence of toxin-induced switching is that herbivores are predicted to spend a disproportionate amount of time foraging on less abundant plant species, resulting in depensatory mortality that can limit invasion by more palatable species (Figure 1). Consistent with this prediction, disproportionate foraging on rare but more palatable food items has been observed in experimental studies with foods that vary in quality [49,50]. Another likely consequence of toxin-determined herbivory is a shift in plant species composition to communities dominated by more toxic plants, as observed in taiga [51,52], southern boreal forest [53], and temperate grasslands [54]. Toxin-determined foraging also may play a role in population cycles of herbivores [55].

## Future directions

Evidence for the importance of plant toxins as determinants of herbivore functional response is indisputable. Recent modeling efforts implicate toxins as potentially key drivers of change in plant communities and herbivore populations. Future models should consider the role of resource patchiness and tri-trophic interactions on plant communities. For instance, adaptive foraging by herbivores is hypothesized to have important effects on ecosystem processes such as nutrient cycling rates, and predators may alter herbivore effects by changing their density or behavior [56]. How do tradeoffs from toxin-induced resource patchiness and risk of predation influence ecosystem properties? From the perspective of evolutionary ecology, models of tradeoffs in plant growth and defense [16] as well as spatio-temporal variation in selection for toxin production [57] may afford greater insight into genetic diversity and geographic structuring of plant populations. At the very least, ecologists conducting work in the future should address explicitly the importance of plant toxins as potential agents of change for plant and herbivore communities.

## Authors' contributions

All authors shared in the conception and organization of this commentary, including review of pertinent literature. RKS wrote the initial draft. All authors read and approved the final manuscript.
